# Supplementary material for: HACSim: an R package to estimate intraspecific sample sizes for genetic diversity assessment using haplotype accumulation curves
Source: PeerJ Comput Sci. 2020 Jan 6;6:e243. doi: 10.7717/peerj-cs.243 (PMC7924493; doi:10.7717/peerj-cs.243)
Supplement: Supplemental Information 4 [file peerj-cs-06-243-s004.pdf]

Supplementary information to accompany `HACSim`: Iterative extrapolation of haplotype accumulation curves for assessment of intraspecific COI DNA barcode diversity estimates

1 Additional Algorithm Parameters

The user also has the ability to subsample data. The function arguments `subset.haps`, `subset.seqs = TRUE`, `prop.haps` and `prop.seqs` are in place to allow for this. A user simply inputs the proportion of haplotype labels (`prop.haps` in the case of hypothetical species) or DNA sequences (`prop.seqs` in the case of real species) to subsample from the entire dataset. Thus, if a species COI alignment comprises $N = 100$ DNA barcode sequences and a subsample of 10% of the data is desired, then a random subset will contain $N = 10$ sequence reads (100×0.10). The effect of subsetting data is that it results in haplotype frequencies being reweighted according to the number of haplotypes contained in the subsample. For instance, if a hypothetical species comprises five unique haplotypes, each with a frequency of 0.20 (20%), and a subsample of four haplotypes is desired (*i.e.*, a proportion of 80% via `prop.haps = 0.80`), then each remaining haplotype in the reduced dataset will have a frequency of 0.25 (25%). A random subsample of haplotypes can be regarded as having been sampled from a single subpopulation/deme in which individuals have migrated into from

neigbouring locations. While this bypasses the challenge of estimating the total number of demes in addition to per-generation migration rates, correlations among sampled populations separated by hundreds to thousands of kilometres will be evident. Hence, in utilizing a subsampling scheme, an important assumption is that all individuals within a given subpopulation are sampled, which may not be the case in reality. Alternatively, a subsampling scheme can be employed to limit the size of large sequence datasets to reduce computation overhead without sacrificing a reduction in the number of permutations (`perms`). Subsampled DNA sequences are automatically written to a FASTA file called 'seqs.fas'.

The function arguments `subsample` and `prop` are used to initialize `subset.haps`, `subset.seqs`, `prop.haps` and `prop.seqs` described previously. These latter four arguments are not used directly by the user. The argument `subsample` takes a binary logical argument of either `TRUE` or `FALSE`, whereas `prop` takes a numeric value between zero and one, exclusive.

Table S1: Algorithm parameters for subsampling haplotype labels or DNA sequences

Parameter	Definition	Range
`subset.haps`	random subset of species haplotypes	(1, H)
`prop.haps`	proportion of haplotypes to subsample	(0, 1)
`subset.seqs`	random subset of species DNA sequences	`TRUE`, `FALSE`
`prop.seqs`	proportion of DNA sequences to subsample	(0, 1)
